# Efficient Production of HIV-1 Virus-Like Particles from a Mammalian Expression Vector Requires the N-Terminal Capsid Domain

**DOI:** 10.1371/journal.pone.0028314

**Published:** 2011-11-30

**Authors:** Pascal Jalaguier, Karine Turcotte, Alexis Danylo, Réjean Cantin, Michel J. Tremblay

**Affiliations:** 1 Centre de Recherche en Infectiologie, Centre Hospitalier Universitaire de Québec – CHUL, Québec, Canada; 2 Département de Microbiologie-Infectiologie et Immunologie, Faculté de médecine, Université Laval, Québec, Canada; Institut National de la Santé et de la Recherche Médicale, France

## Abstract

It is now well accepted that the structural protein Pr55^Gag^ is sufficient by itself to produce HIV-1 virus-like particles (VLPs). This polyprotein precursor contains different domains including matrix, capsid, SP1, nucleocapsid, SP2 and p6. In the present study, we wanted to determine by mutagenesis which region(s) is essential to the production of VLPs when Pr55^Gag^ is inserted in a mammalian expression vector, which allows studying the protein of interest in the absence of other viral proteins. To do so, we first studied a minimal Pr55^Gag^ sequence called Gag min that was used previously. We found that Gag min fails to produce VLPs when expressed in an expression vector instead of within a molecular clone. This failure occurs early in the cell at the assembly of viral proteins. We then generated a series of deletion and substitution mutants, and examined their ability to produce VLPs by combining biochemical and microscopic approaches. We demonstrate that the matrix region is not necessary, but that the efficiency of VLP production depends strongly on the presence of its basic region. Moreover, the presence of the N-terminal domain of capsid is required for VLP production when Gag is expressed alone. These findings, combined with previous observations indicating that HIV-1 Pr55^Gag^-derived VLPs act as potent stimulators of innate and acquired immunity, make the use of this strategy worth considering for vaccine development.

## Introduction

Virus-like particles (VLPs) have sparked an increasing interest over the last decade [1], [2]. These viral entities cannot perform a full replication cycle and therefore cannot produce new progeny virus. This property has allowed them to be used and studied in different contexts. They serve notably in gene therapy investigations, as they can enter target cells and deliver a specific gene [3]. They are also employed for the study of chemical compounds that can block particle assembly and could prove to be useful in antiviral therapies. Finally, VLPs have recently been used with success as a tool for vaccination against human papillomavirus [4], [5], [6]. These recent successes underline the value of deepening our understanding of the mechanisms involved in VLP formation for many viruses, including human immunodeficiency virus type-1 (HIV-1).

HIV-1 can form VLPs by sole virtue of its Gag polyprotein precursor, also known as Pr55^Gag^. This structural viral constituent is indeed the main actor in HIV-1 particle formation as it drives assembly through protein-protein, protein-RNA, and protein-lipid interactions, orchestrating the incorporation of each of the major virus-encoded components into assembled particles [7], [8], [9], [10]. Pr55^Gag^ is translated from unspliced viral mRNA on free ribosomes in the cytoplasm and encodes the internal structural components of the virion, i.e. matrix (MA), capsid (CA) and nucleocapsid (NC) along with the C-terminal p6 domain and two spacer peptides SP1 and SP2 [11]. In the absence of any other viral proteins, Pr55^Gag^ can self-assemble into VLPs [12] by ordered multimerization of monomers and/or oligomers to produce a spherical shell, which forms the structural framework of the immature virus particle [10], [13]. It is noteworthy that HIV-1 Pr55^Gag^-based VLPs elicit strong cellular and humoral responses in non-human primate research models for AIDS [1], [14], [15].

Several regions of the Pr55^Gag^ polyprotein have been reported to be crucial for particle formation. Notably, previous structural and genetic analyses have demonstrated that the regions responsible for membrane binding are located at the N-terminal region of MA, which encompasses a cluster of basic residues and a myristyl group interacting with acidic phospholipids of the membrane [16], [17]. However, several studies have shown that MA can be largely deleted or even entirely replaced by a heterologous myristyl anchor without compromising the formation of extracellular viruses [18], [19], [20], [21]. This suggests that the role of MA in assembly is not as important as its role in Pr55^Gag^ trafficking [22], [23] and in the incorporation of virus-encoded envelope glycoproteins (ENV) [24], [25].

CA provides structural stability to HIV-1 particles and also plays a key role in the formation of protein-protein contacts required for a productive particle assembly. CA comprises two globular domains: an N-terminal domain (NTD) and a C-terminal domain (CTD) linked by a short flexible sequence [10], [26], [27]. The NTD consists of five long helices forming a stable coiled-coil structure, two more short helices, two hairpins, and a proline-rich loop. NTD is considered to play a structural role in core formation rather than actively driving particle production [18], [19]. Indeed, Gag lacking this entire domain was shown to assemble and bud from the host cell [18], [19]. However, previous studies have shown that Gag constructs bearing certain specific mutations or in-frame insertions in the N-terminal domain gave rise to a dramatically reduced particle production even in the absence of the other viral components, such as genomic RNA, envelope glycoproteins, enzymes and auxillary proteins [28], [29], [30], [31]. The authors identified a previously unrecognized contiguous CA domain located beneath the cyclophilin-A binding loop that participates in Gag assembly. In contrast, CTD is recognized as being fundamental for VLP formation. This specific region contains four conserved helices and a highly conserved sequence among retroviruses known as the major homology region (MHR) [11]. The MHR determines the Gag conformation required for efficient protein-protein and protein-membrane interactions during virus assembly [31], [32], [33]. Moreover, the hypothetical α-helix formed by the last residues of CTD and SP1 participates in the assembly process [34]. Mutations within the 14-amino acid peptide that separates CA from NC or SP1 have been reported to induce strongly aberrant budding structures and reduce the number of released extracellular particles [31], [35], [36], [37].

NC contains two zinc finger domains that bind RNA, and an interaction domain (I domain), consisting in a stretch of basic residues, that mediates Gag-Gag interactions [7], [38]. Interestingly, the zinc fingers are not absolutely necessary for the function of the I domain [38], [39], [40]. Nevertheless, the role of RNA in assembly is so vital that HIV-1 will recruit other cellular RNAs by default if viral RNA is not available [41]. As far as the assembly process is concerned though, NC can be efficiently replaced by a heterologous leucine zipper (LZ) domain (i.e. a coiled-coil sequence capable of forming parallel homodimers) [18], [42], [43].

The p6 domain contains two late domains (LDs), which are required for pinching off the newly assembled virions from the host membrane. The first one, located in N-terminal position, is the principal LD. It contains a proline-rich PTAP motif, which appears to play a critical role in exocytosis of assembled particles [44]. PTAP interacts directly with Tsg101 [45], which is a host component of the endosomal sorting complex required for transport type-I (ESCRT-I) [46]. The second LD region located in C-terminal contains a YPLTSL motif that interacts with another cellular machinery molecule, i.e. AIP-1/ALIX (link between ESCRT-I, and –II) [47]. It is well established that, in some conditions, p6 can be replaced with a heterologous LD sequence, notably with p2b, a small spacer peptide from another retrovirus, i.e. Rous Sarcoma Virus (RSV), containing the PPPY motif [18], [48], [49].

Although there are numerous bacterial, yeast and insect systems to produce HIV-1-based VLP [1], [2], [15], the vast majority of VLP studies using a mammalian system have been performed in a provirus context [19], [21], [43]. Very few studies used solely the Pr55^Gag^ polyprotein under the control of another promoter than LTR, and obviously in the absence of virus auxiliary proteins. The latter conditions moreover correspond to the common setup for vaccine studies. Now, Accola and colleagues previously described a minimal sequence of Pr55^Gag^ able to produce VLPs as efficiently as wild type virus [18]. This chimera protein, expressed as part of a molecular clone of HIV-1, was shown to be efficiently released from host cells. In the present study, we show that a similar minimal sequence can result in different experimental observations when inserted in a mammalian expression vector instead of being part of a molecular clone. Given this discrepancy, we investigated further and, to do so, designed a series of Pr55^Gag^ mutants with the aim of identifying which region(s) of Pr55^Gag^ are necessary to VLP formation in our experimental approach. We examined expression and VLP formation of these mutants by biochemical and electron microscopy approaches and, in agreement with Accola and co-workers, we demonstrate that MA is not necessary, and that NC and p6 are dispensable when heterologous sequences like hCREB and p2b are used. However, we observed that, in the context of a mammalian expression vector, VLP formation requires the presence of the NTD of the CA domain (amino acids 133 to 277) for assembly, budding and release in human human embryonic kidney 293T (HEK293T) cells.

## Results

We tested in this study the VLP-producing ability of a minimal Pr55^Gag^ sequence inserted in a strong mammalian expression vector, namely pcDNA3.1 (+). One of the major advantages of a plasmid over a molecular clone for producing VLPs is that, most evidently, the particles produced do not replicate, cannot revert to an infectious virus, and are thus much safer. We decided to evaluate a minimal Gag construct (called Gag min hereinafter) that had already been reported in the literature by Accola and colleagues [18]. This mutant was derived from the HXBH10-PR^-^ molecular clone, which is identical to HXBH10, a Vpu-positive variant of the HXB2 HIV-1 molecular clone, except for a point mutation that inactivates the protease (PR) [18]. The chimera protein was shown to be efficiently released from cells. It comprises only 6 amino acids of the myristoylation signal in MA, only residues 146 to 231 of CA, the 14 amino acids of SP1, and heterologous sequences that replace NC and p6.

**Figure 1:**
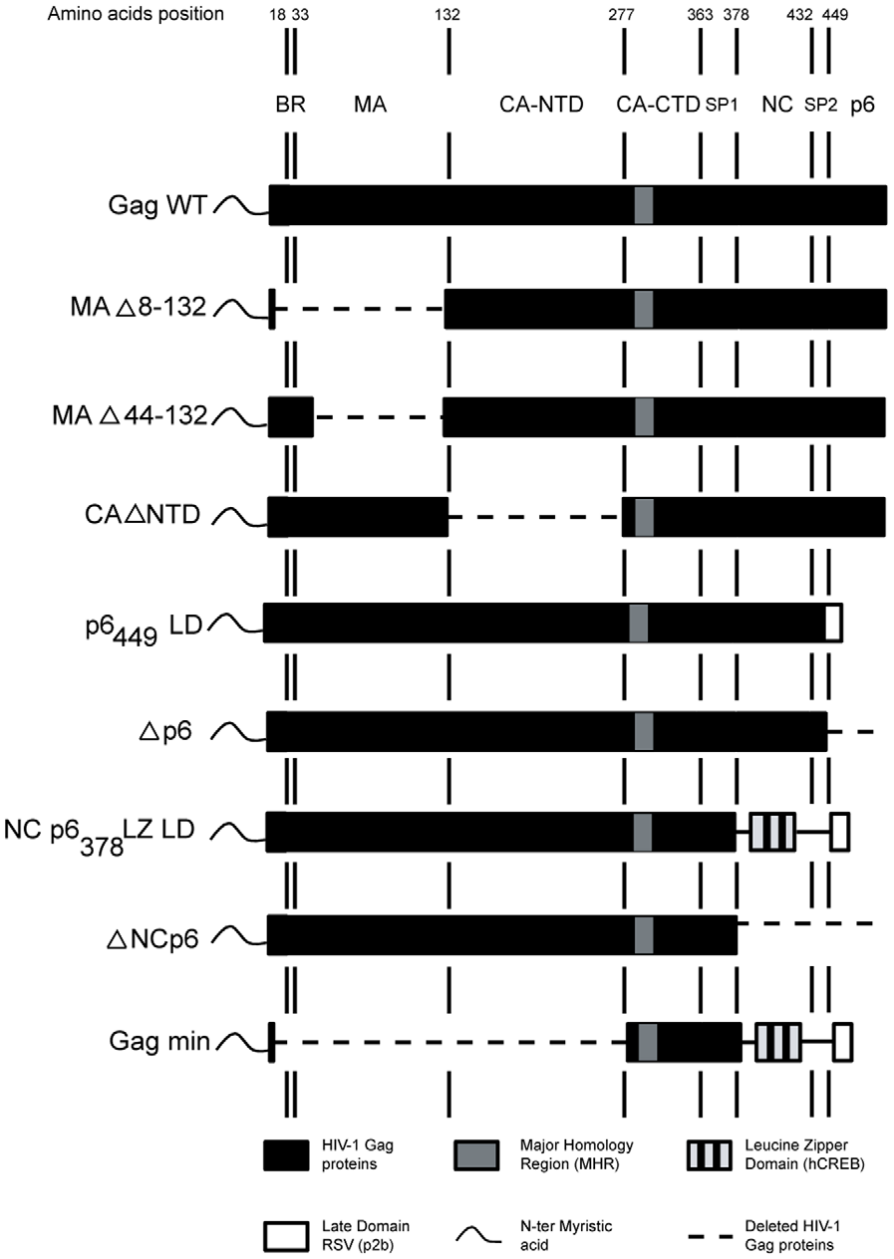
Schematic representation of the different HIV-1 Pr55^Gag^-derived constructs used in this study. The various molecular constructs, derived from the HXB2 Pr55^Gag^ sequence (designated here as Gag WT), were made by PCR as described under the *Materials and Methods* section. All constructs contain the wild-type myristic acid post-translational sequence in the N-terminal position and all were subcloned into the pcDNA3.1 (+) eukaryotic expression vector. Wild-type HIV-1 sequences are denoted in black, and deleted virus sequences correspond to the dotted line. Numbers above correspond to amino acids in the wild-type sequence. BR, basic region of MA; NTD, N-terminal domain; CTD, C-terminal domain.

As illustrated in Fig. 1 (bottom line), our Gag min construct is identical to the one previously described [18], except for the heterologous NC sequence, which in our case consists in the hCREB-1 sequence. This sequence forms a coiled-coil structure capable of forming parallel homodimers. We decided to use this basic LZ domain (bZIP) sequence instead of the yeast-derived GNC4 sequence used previously [18] because, when formulating a vaccine, one should avoid including foreign sequences in order not to induce non-specific cross-immune reactions. Moreover, the hCREB-1 sequence was successfully used in the past for the same purpose [43]. Finally, to improve the efficiency of protein translation in HEK293T cells, codons were humanized [50].

### Gag min fails to produce VLPs

**Figure 2:**
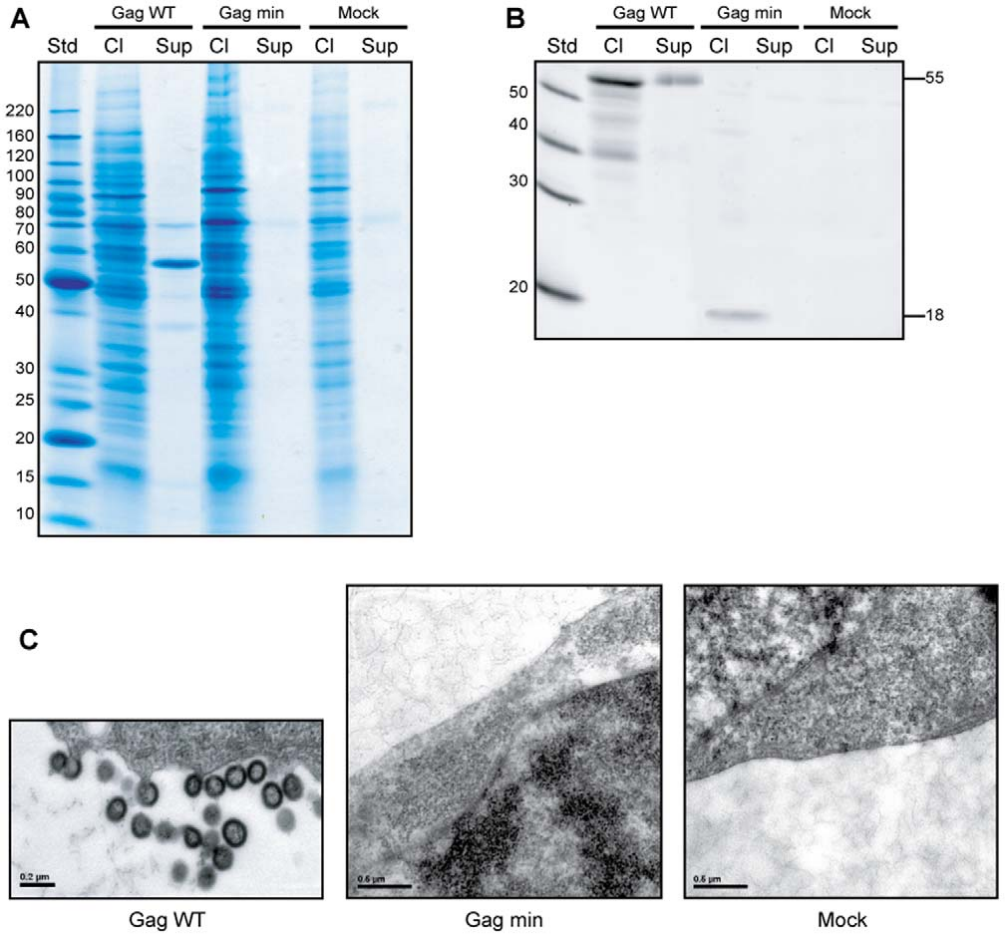
Gag min in the context of an expression vector fails to produce VLPs. (A) 293T cells were transfected with vectors expressing either Gag WT or Gag min. The mock represents 293T cells transfected with the empty pcDNA3.1 (+) vector (used as a negative control). At 48 h post-transfection, cell lysates (Cl) and cell-free supernatants (Sup) were analyzed on a gradient SDS-PAGE and the protein stained with Coomassie Brilliant Blue. (B) In parallel, an IP assay was performed using an anti-p24 antibody on the same samples (Cl and Sup). Precipitated proteins were run on a gradient SDS-PAGE and Gag proteins were analyzed by western blotting (WB) with a biotinylated anti-p24 antibody. (C) 293T cells expressing Gag WT or Gag min construct were analyzed by TEM (magnification: × 50,000). The Biochemical experiments shown are representative of at least three independent experiments and two for the microphotographs.

To determine how Gag min behaves in our VLP experimental model system, this vector was first transiently transfected in HEK293T cells as well Gag WT (used as a positive control). At 48 h post-transfection, cell-free supernatants containing VLPs were harvested and transfected cells were collected as well, and prepared as described in *Methods* section. Both VLPs and cell lysates were analyzed on a gradient SDS-PAGE, and the gel was next stained with Coomassie blue. As depicted in Fig. 2A, a major band at a molecular weight (MW) of 55 kDa was observed for Gag WT and, as expected, no similar protein signal appeared in the cell-free supernatant from cells transfected with an empty control vector (called mock). However, we failed to detect a band at the predicted MW of Gag min (theoretical MW (MW_th_) = 18.2 kDa) in the cell-free supernatant. Too many bands were present in cell lysis samples to allow identifying the different proteins of interest. To achieve better sensibility and specificity, we performed immunoprecipitation (IP) assays on the same samples with an anti-p24 antibody, followed by western blot detection. With this technical strategy, we obtained for Gag min a specific signal at the predicted 18.2 kDa in the cell lysate (Fig. 2B). However, we were still unable to detect a signal in the cell-free supernatant, whereas we detected a distinctive band for the positive Gag WT control.

At this point, we hypothesized that the Gag min protein was being expressed by the transfected cell, but could not give rise to VLP production due, for example, to a malfunction in the assembly process. To explore this possibility, we analyzed our transiently-transfected HEK293T cells by transmission electron microscopy (TEM). For Gag min, we did not observe any VLP in the process of budding through the cell membrane, or in the supernatant close to the cell, in accordance with our previous biochemical experiments (Fig. 2C). Moreover, there was no VLP, whether incomplete or fully formed, in the cytoplasm of the transiently transfected cells. This is in sharp contrast to Gag WT that showed a characteristic VLP spherical morphology (average size around 100 nm), similar to that of immature HIV-1 particles [51].

**Figure 3:**
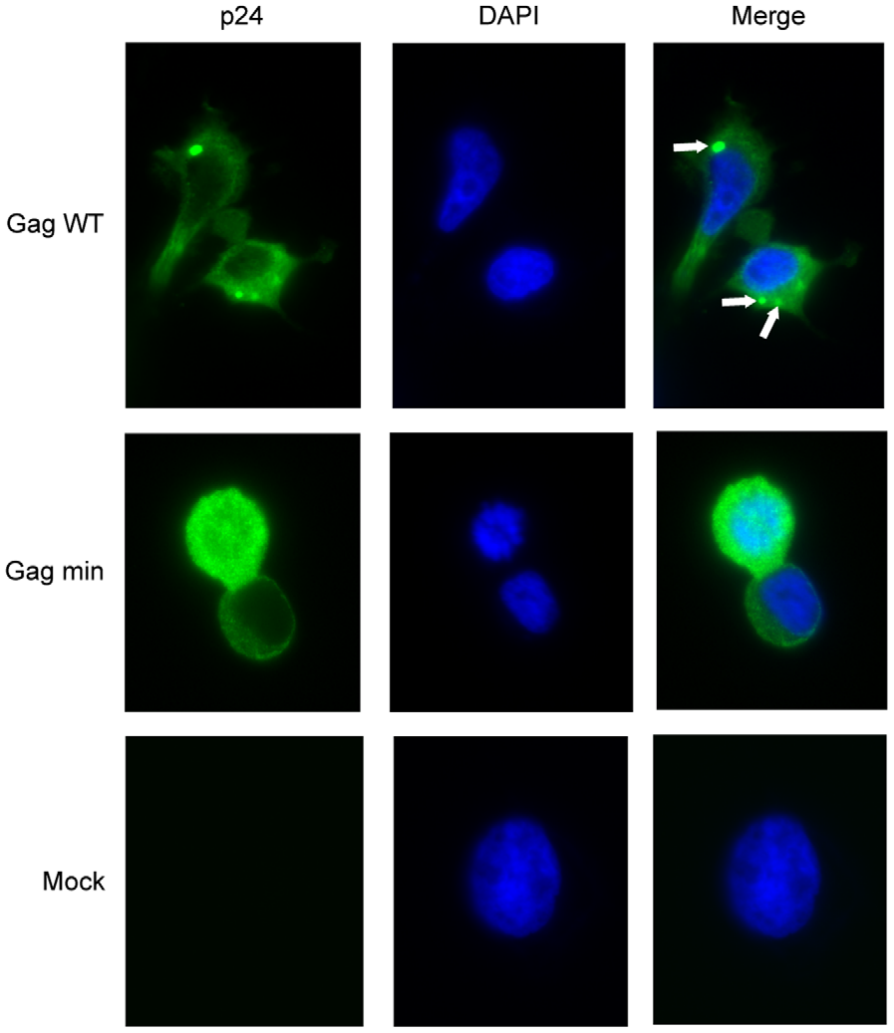
Gag min proteins show a diffuse distribution pattern. 293T cells were transfected either with Gag WT (upper panels), Gag min (middle panels) or pcDNA3.1 (+) vector (bottom panels). At 48 h post-transfection, cells were fixed and permeabilized for immunofluorescence assays as described in *Materials and Methods*. Nuclei were stained with DAPI. White arrows indicate a punctiform distribution of Gag proteins. The samples were visualized with a Nikon eclipse TE 300 inverted microscope (magnification: x 1,000). The experiment shown is representative of 3 independent experiments.

To further investigate the intracellular location of the Gag min proteins within the transfected cells, indirect immunofluorescence studies were performed with an anti-p24 antibody. As illustrated in Fig. 3 (upper panel), Gag WT proteins were detected throughout the cytoplasm of transfected cells, organized in dotted structures suggesting they were undergoing VLP assembly. In contrast, cells transfected with the Gag min vector showed a more diffuse distribution pattern of proteins within the cytoplasm (middle panels). Thus, both biochemical and microscopy studies clearly show that proteins expressed upon transfection of the Gag min construct do not assemble properly when inserted within an expression vector.

We next determine why this Gag min construct fails to form VLPs in our experimental setting. Considering that it was designed through investigations that almost always used full-length molecular clones [18], [19], [21], we hypothesized that different sequence stretches may be required for proper assembly of Gag in the absence of all other viral proteins. We thus produced a series of deletion mutants (Fig. 1), to re-examine the necessity of the various Gag domains for producing VLPs, in the context of an expression vector.

### MA is not required for VLP production, but the basic region allows a more efficient particle release

**Figure 4:**
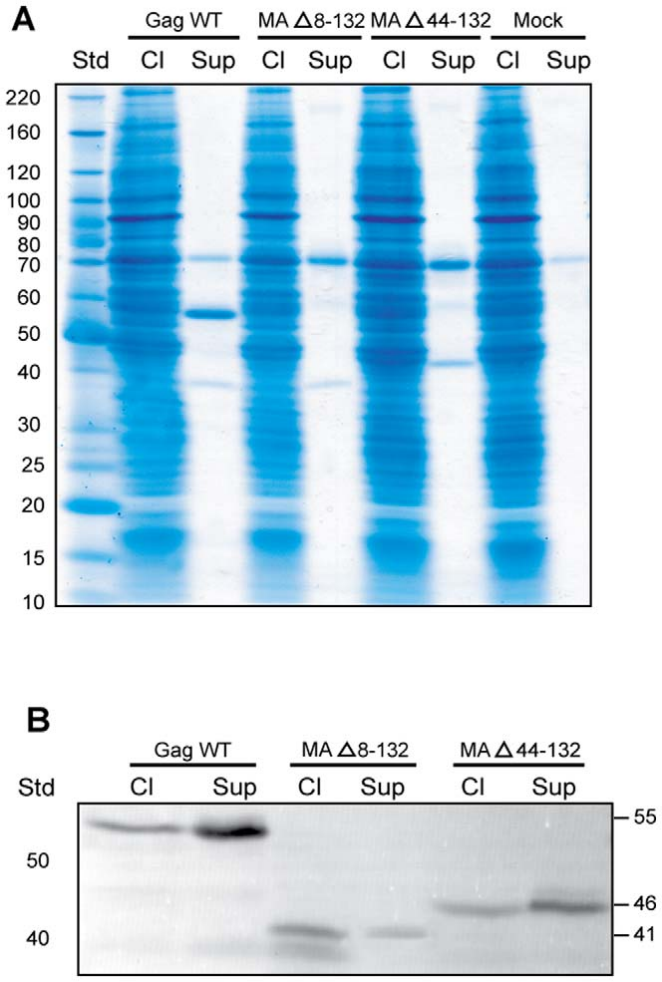
MA region is not required for VLP production. (A) 293T cells were transfected either with Gag WT, MA mutants (i.e. MAΔ8–132 and MAΔ44–132) or pcDNA3.1 (+) vector. At 48 h post-transfection, both the cell lysates and cell-free supernatants were analyzed on a gradient SDS-PAGE with Coomassie Brilliant Blue staining. (B) In parallel, an IP assay was performed using the anti-p24 antibody on the same samples. Precipitated proteins were run on gradient SDS-PAGE and Gag proteins were analyzed by Western Blotting (WB). The Biochemical experiments shown are representative of at least three independent experiments.

**Figure 5:**
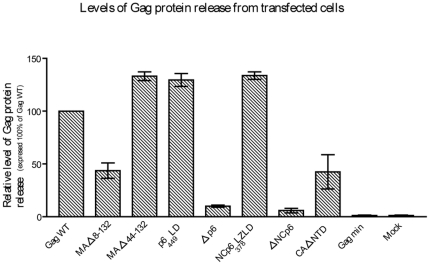
Quantitative analysis of VLP formation. Gag protein signals from cell-free supernatants and cell lysates following transfection with the studied HIV-1 Pr55^Gag^-derived plasmids were quantified by scanning the band densities on the WB membranes using a Typhoon 9200 fluorescent laser scanner. Ratios of total Gag protein levels in the cell-free supernatants to those in cells extracts were determined for each construct, and compared with the same ratio for Gag WT. Results shown were obtained by dividing the release ratio for each mutant by the ratio for the Gag WT and multiplying by 100. Data shown represent at least three independent experiments for each mutant. Error bars indicate standard deviations.

It is now well established that the MA region plays both structural and targeting roles in the virus assembly process, but appears to be somewhat less important in Gag multimerization. The latter phenomenon relies largely on two key sequences. First, the N-terminal myristyl acid has been demonstrated to interact with the inner leaflet of the plasma membrane by hydrophobic interactions [16], [52], [53], [54]. Its absence prevents particle release [22], [23], [55]. Second, the basic region (BR) (amino acids 18 to 33), which is also located in the N-terminal position, plays a pleiotropic role in the assembly process, notably in plasma membrane targeting and binding. We thus produced two MA mutants differing by the presence (MAΔ44–132) or the absence of BR (MAΔ8–132) in order to investigate whether BR is required to allow formation of VLPs. We detected the MA deletant proteins in both the cell lysates and cell-free supernatants. Those proteins were detected either with SDS-PAGE (Fig. 4A) or following immunoprecipitation with an anti-p24 antibody (Fig. 4B). The bands detected at 41 and 46 kDa respectively match the expected molecular weight of MAΔ8–132 (Mw_th_ = 41.72 kDa) and MAΔ44–132 (Mw_th_ = 46.02 kDa). However, the efficiency of particle release proved to be different between these two mutants. Indeed, MAΔ8–132 released about half as many particles as Gag WT, whereas MAΔ44–132 released around 40% more than Gag WT (Fig. 5). In other words, there is approximately a 3-fold difference between these two mutants. Please note that, for comparison purposes, Fig. 5 shows the relative efficiency of release of VLPs for all mutants assayed in this study.

**Figure 6:**
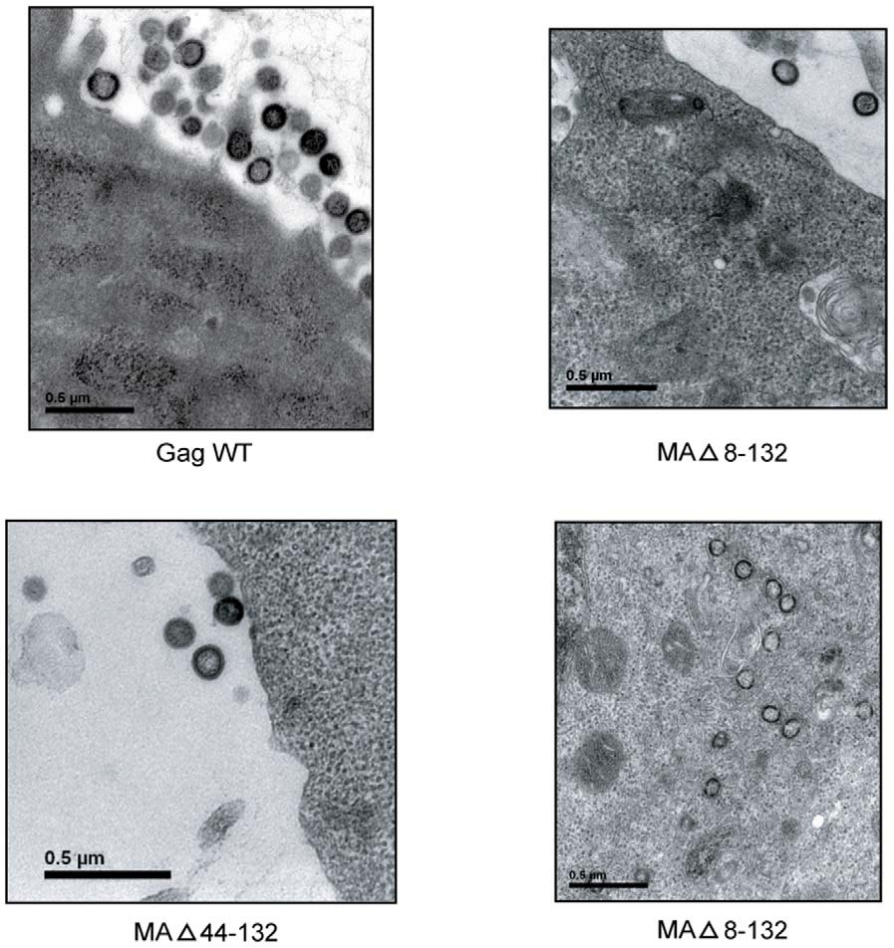
The Basic Region of MA allows a more efficient particle release. 293T cells expressing either Gag WT or MA mutants (i.e. MAΔ8–132 and MAΔ44–132) were examined by TEM (magnification: × 50,000). The microphotographs shown are representative of 2 independent experiments.

Subsequently, to visually confirm the results described above and to examine the site of intracellular particle formation of the two MA mutants, we performed TEM on HEK293T cells expressing Gag WT, MAΔ8–132 and MAΔ44–132. An empty control vector was also tested as well (data not shown). First, morphological analysis of the two MA mutants confirmed their typical VLP shape with spherical-like morphology and electron-dense ENV (Fig. 6). In addition, for the MAΔ8–132 VLPs we observed many points of intracellular assembly inside the cytoplasm (bottom right). We however could not see this phenomenon with the MAΔ44–132 VLPs, but rather more numerous particles in the extracellular space (bottom left). These observations confirm the discrepancy in particle release between these two MA mutants. Globally, the results above show that the MA domain can be dispensable for VLP formation, although the basic region seems to promote particle production efficiency.

### NC and p6 regions are required for VLP release, although they can be replaced with heterologous sequences hCREB and p2b

**Figure 7:**
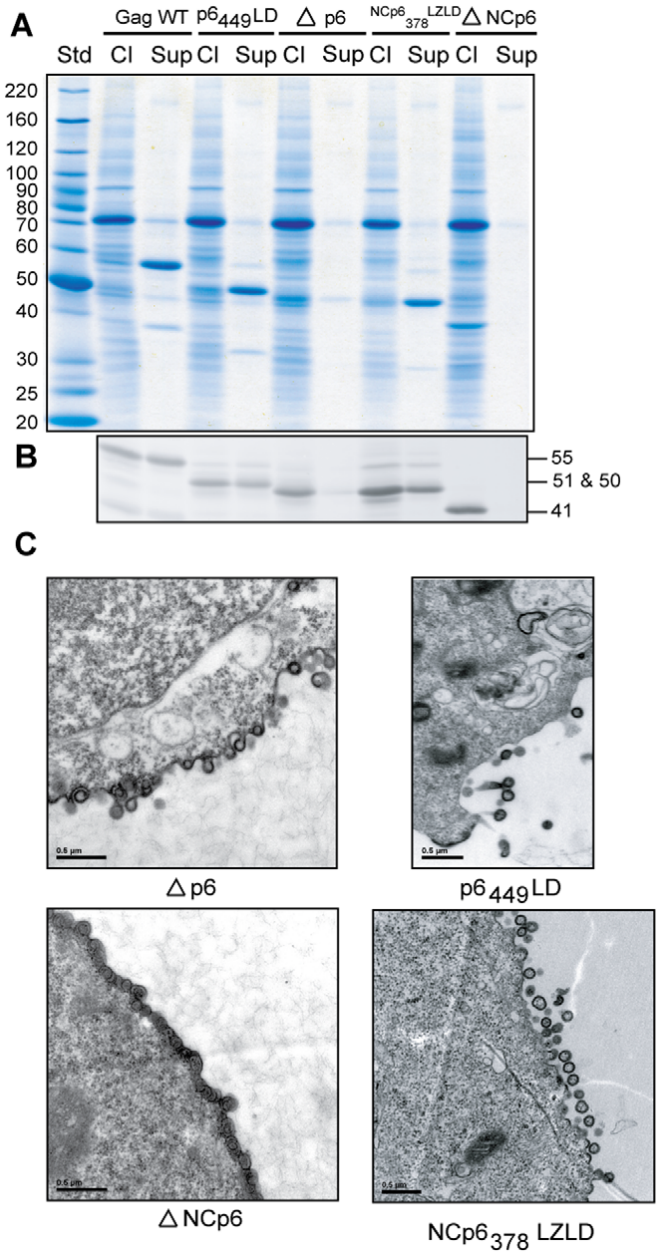
Domains NC and p6 are required for VLP release, although they can be replaced with heterologous hCREB and p2b sequences. (A) 293T cells were transfected either with Gag WT or expression vectors encoding for NC or P6 mutants (i.e. Δp6, ΔNCp6, p6_449_LD and NCp6_378_LZLD). At 48 h post-transfection, cell lysates and cell-free supernatants were analyzed on gradient SDS-PAGE and protein stained with Coomassie Brilliant Blue. (B) In parallel, an IP assay was performed using the anti-p24 antibody on the same samples. Precipitated proteins were run on SDS-PAGE and Gag proteins were analyzed by WB. (C) Transfected 293T cells were analyzed by TEM (magnification: × 50,000). The Biochemical experiments shown are representative of at least three independent experiments and two for the microphotographs.

We next investigated the possible contribution of NC and p6 domains in VLP formation. According to the literature, the p6 domain is important in the final release process from infected cells, whereas NC participates in Gag-Gag interactions [46], [56]. We thus designed the deletion mutants Δp6 and ΔNCp6 as illustrated in Fig. 1. As with VLPs produced from molecular clones [42], [43], the NC and p6 deletion constructs were unable to produce VLPs. Indeed, cell-free supernatant analysis of transfected HEK293T cells did not permit to detect any protein signal, be it with SDS-PAGE (Fig. 7A) or following an IP/western blot assay (Fig. 7B). However, the two deletion mutants were clearly expressed because we were able to detect protein signals in cell lysate fractions. Respective molecular weights of 41 and 50 kDa were detected for ΔNCp6 (Mw_th_ = 41.91 kDa) and Δp6 (Mw_th_ = 50.14 kDa). Results from TEM analyses allowed us to confirm a default in VLP release for those two different constructs (Fig. 7C) (data not shown for the Gag WT control). The Δp6 mutant shows a pinching-off default, as VLPs remain tethered to the cell plasma membrane. On the other hand, ΔNCp6 showed an intriguing morphology. Indeed, VLP formation and assembly occur close to the inner leaflet of the plasma membrane, but the particles seem to accumulate at the membrane in large quantities, while showing no sign of pinching-off (Fig. 7C, bottom left).

The above results confirm that p6 and NC are necessary for our VLP model. Moreover, we evaluated if heterologous sequences were able to efficiently substitute NC and p6 domains, like they can when Gag is expressed in a molecular clone [18]. We designed 2 substitution mutants, p6_449_LD and NCp6_378_LZLD. Concerning p6_449_LD, this construct differs from the Gag WT only by the substitution of the p6 domain with the RSV p2b sequence (see introduction section for more details). Overall, p6_449_LD was expressed intracellularly and produced VLPs to a similar extent as the Gag WT (release efficiency of about 1.4 fold compared to Gag WT) (Fig. 5). The p6_449_LD protein signal was detected both by SDS-PAGE and western blot assays (Figs. 7A and 7B) with a 50 kDa molecular weight (Mw_th_ = 51.18 kDa). TEM data showed that the morphology of p6_449_LD VLPs (Fig. 7C, upper right panel) is comparable to that of the Gag WT (data not shown).

Next, to get closer to the structure of the Gag min construct, we performed a double substitution where, in addition to replacing p6 with p2b, we replaced NC and SP2 by hCREB-1. As expected, the resulting NCp6_378_LZLD mutant was able to generate VLPs, which were released efficiently from cells (Figs. 7A and 7 B). This was confirmed by TEM (Fig. 7C, bottom right) (1.4 fold compared to Gag WT) (Fig. 5). Taken together, the above substitutions and deletions in our VLP model confirm that both NC and p6 are required for particle release. In addition, their substitution with heterologous sequences does not diminish the efficiency of the Gag plasmid.

### N-terminal domain of CA is necessary for VLP formation

**Figure 8:**
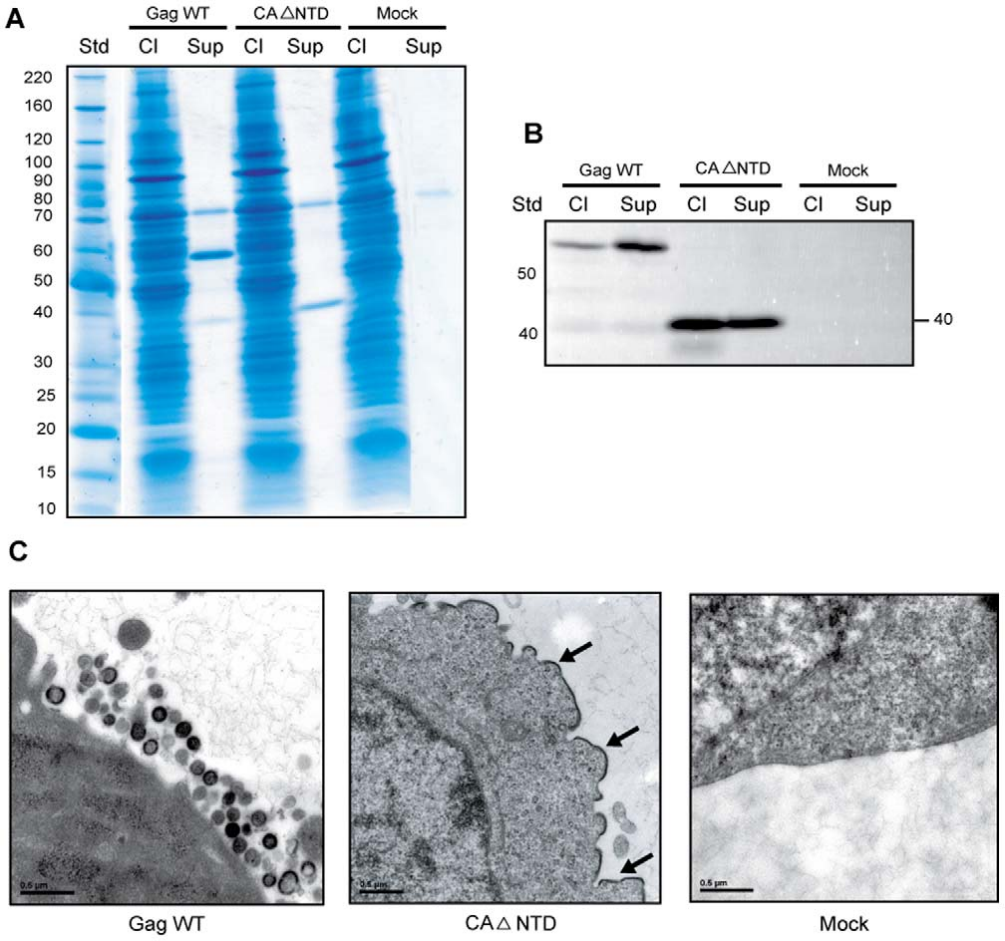
Domains NC and p6 are required for VLP release, although they can be replaced with heterologous hCREB and p2b sequences. (A) 293T cells were transfected either with Gag WT or expression vectors encoding for NC or P6 mutants (i.e. Δp6, ΔNCp6, p6_449_LD and NCp6_378_LZLD). At 48 h post-transfection, cell lysates and cell-free supernatants were analyzed on gradient SDS-PAGE and protein stained with Coomassie Brilliant Blue. (B) In parallel, an IP assay was performed using the anti-p24 antibody on the same samples. Precipitated proteins were run on SDS-PAGE and Gag proteins were analyzed by WB. (C) Transfected 293T cells were analyzed by TEM (magnification: × 50,000). The Biochemical experiments shown are representative of at least three independent experiments and two for the microphotographs.

The last domain remaining to be investigated was the CA protein. We focused our attention on the NTD because the CTD has been recognized as essential to the particle assembly process. On the other hand, controversial results have been obtained with NTD, as explained in the introduction section. Thus, we designed a CA NTD mutant where the complete NTD region (amino acids 133 to 277) was deleted like in the Gag min mutant. This new mutant named CAΔNTD at first sight appeared to be able to produce VLPs efficiently as shown both by SDS-PAGE and IP/western blot (Figs. 8A and 8B). However, in contrast to previous studies, the morphological characterization failed to confirm that our protein gel results correspond to bona fide VLP. In fact, this mutant showed a severe default in the assembly and budding process (Fig. 8C). Nevertheless, this finding is consistent with previous observations made in baculovirus-infected cells by Chazal and colleagues where a Gag insertion mutant at position 209 resulted in a very similar cellular membrane phenotype [28]. Our CAΔNTD mutant actually shows very large electron-dense patches of the Gag mutant protein at deformed areas of the plasma membrane.

In conclusion, the CAΔNTD mutant was unable to form VLPs in our hands. We hypothesize that the supernatant protein signals detected biochemically may correspond partially to cell-free patches of cell plasma membrane containing densely-aggregated Gag mutant proteins.

## Discussion

In the present work, we designed a series of Pr55^Gag^ mutants with the aim of defining, in the context of a mammalian expression vector, the intrinsic role of the Gag protein in the assembly and release of immature VLPs from human producer cells. Past investigations almost always used full-length molecular clones in which a point mutation inactivated the viral protease gene in order to produce immature HIV-1 particles [18], [19], [21]. However, using these molecular constructs, one cannot exclude the influence on the assembly process of other viral gene components like ENV or accessory molecules. Those viral accessory proteins are not desirable when investigating Gag auto-assembly. Also, working with mammalian expression plasmids presents a number of other advantages, as compared to a full-length molecular clone. For example, smaller plasmids are easier to handle. More importantly, the particles produced have no possibility of replication and cannot revert to an infectious virus. We thus studied VLP formation in the complete absence of the other viral proteins, in the perspective of gathering valuable information for future vaccine design.

To achieve this goal, we expressed the precursor Pr55^Gag^ polyprotein under a promoter different from the HIV-1 LTR in a mammalian expression vector. On the basis of biochemical and microscopical data, we found that, in contrast to previous published observations [18], the minimal Gag chimera protein failed to produce VLPs. In fact, we were unable to detect viral protein in the supernatant, nor any budding structures in TEM studies. Nevertheless, the chimera protein is expressed inside the cells, because we were able to detect a signal by western blot and indirect immunofluorescence microscopy (Figs. 2 and 3). In addition, we detected the mRNA by RT-PCR in cell extracts (data not shown). The failure seems to happen early in the assembly process, as illustrated by the very diffuse signal appearing in microphotographs (Fig. 3). It could consist in a major preassembly default. This sharply contrasts with the Gag WT proteins that display a punctate staining pattern, a phenotype in accordance with previous studies [57], [58], [59], [60]. These sites of intense Gag localization presumably represent active centers of virus assembly. Characterization of such sites will provide additional insights into the mechanism of Gag trafficking and virus assembly.

Our results though do not necessarily contradict those of Accola and colleagues [18], as the noted discrepancies can be explained by numerous factors. First, the authors worked with a different expression system, which was based on the HXBH10-PR^-^ molecular clone, and, as stated above, the presence of all virus proteins may influence the action of the Gag protein during VLP production. Second, we used a different dimerization domain since we substituted the previously used GCN4 sequence by the hCREB leucine zipper domain. Although this sequence had already proven to efficiently replace NC in a previous work [43], we cannot rule out the possibility that hCREB and GCN4 differently affect VLP formation. However, it must be noted that the substitution of NC by hCREB did not modify VLP production in our assays. Third, Accola and co-workers used only biochemical approaches in their study. They did not provide any electron microscopic studies in their work. Therefore, a direct visual comparison with our observations is lacking. Most importantly, it is our TEM studies that allowed us to detect a default in assembly and budding of the CAΔNTD mutant, whereas biochemical approaches indicated VLP production. Fourth, the authors used different methods for detecting VLPs in cell lysates and in supernatants; for the latter, they used ^35^[S] methionine metabolic labelling, a method well-known for its sensitivity, whereas they performed a classical WB on cell lysates. On the other hand, we revealed the Gag protein with a biotinylated monoclonal antibody against p24 and the primary antibody was detected with Cy-3-conjugated streptavidin technology both for cell lysates and VLP with the aim of keeping the same detection method for the two types of samples.

In our quest to determine the reason(s) why Gag min does not produce VLPs, we designed a series of mutants/deletants derived from Gag WT and Gag min in order to find out which domain(s) appears to be essential to produce VLPs in our conditions. We confirmed that, at least under our experimental conditions, the MA domain is dispensable for VLP formation as long as two fundamental elements are present: (1) the myristyl acid in the MA N-terminal position that interacts with the inner leaflet of the plasma membrane by hydrophobic interactions [16], [52], [53], [61]. The absence of this myristyl acid prevents particle release [22], [23], [55]; (2) the basic region (BR: 18–33) in the MA N-terminal position, that fulfills multiple functions in assembly process, like plasma membrane targeting and binding [54], [59], [61], [62]. Indeed, MAΔ44–132 is efficiently produced like depicted in Fig. 4 and is as efficiently released as Gag WT.

In comparison, the MAΔ8–132 mutant, in which almost the totality of MA was deleted, is still able to produce VLPs, but less efficiently than Gag WT or MAΔ44–132. We observed through microscopy that this mutant lacking the BR region presents partial virus assembly and that assembly is redirected from the plasma membrane to intracellular membranes (Fig. 6) [54], [61], [63]. More precisely, we can suggest here that, like Ono *et al.* have already reported [33], assembly of BR-deficient mutant partially takes place in Golgi or post Golgi vesicles.

Concerning the NC and p6 substitutions, we are in agreement with previous studies that demonstrated the importance of these two domains for VLP production. It seems clear that the deletion of p6 results in severe defaults in VLP release, which is in agreement with the literature [44]. Although the Δp6 mutant displays a classical VLP morphology, assembling at the plasma membrane, the particles appear unable to pinch off the cell membrane. They seem to remain tethered to the cell surface, which implies the necessity of a late domain that is able to interact with ESCRT complexes for efficiently budding out of the cells [8], [9], [46]. On the other hand, we obtained for the ΔNCp6 mutant results somewhat different from previously published ones. As depicted in Fig. 7C, Gag seems to assemble directly underneath the plasma membrane, in contrast to the formation of large patches of Gag already described [42]. Considering that the ΔNCp6 mutant consists in MA, CA and SP1, it is not surprising that the targeting of the protein to the plasma membrane is occurring. The CA region, along with the adjoining SP1 14 amino acids, may adopt a hypothetical α-helix conformation. This region is well known to mediate strong Gag-Gag interactions, leading to higher-order multimerization critical for particle assembly. Therefore, the morphology observed in this TEM study for the ΔNCp6 mutant agrees with its expected properties.

Now, concerning the double substitution mutant NCp6_378_LZLD, it is released efficiently from HEK293T cells as confirmed by the TEM results. This agrees with previous substitutions of p6 with the RSV p2b region [48], and of NC with a heterologous LZ domain [43]. It is noteworthy that for replacing NC, instead of the GCN4 yeast sequence used in Gag min by Accola *et al.* we selected the hCREB sequence, a sequence successfully used previously for the same purpose [43]. Indeed, in the perspective of the VLPs being used in vaccine preparations, we preferred to avoid including an exogenous domain, which can interfere with the immune VLP specific response. Moreover, to the best of our knowledge, this is the first such double substitution of NC and p6, in an otherwise intact Gag.

Finally, the most noteworthy mutant in this study is CAΔNTD, in which the N-terminal domain is entirely deleted. With biochemical methods, we were able to detect, as expected, a protein signal for CAΔNTD, both in the cell lysate and supernatant, indicating that this mutant was able to produce VLPs. Remarkably however, electron microscopy showed a total absence of VLP formation, either intracellularly or extracellularly (Fig. 8C). We suggest that the supernatant protein signals detected biochemically may correspond partially to cell-free patches of cell plasma membrane containing densely-aggregated Gag mutant proteins and not bona fide VLPs. However the cellular release of microvesicles and/or exosomes cannot be totally excluded even though this scenario is not supported by our TEM data. Additional studies are warranted to validate this postulate. This discovery disagrees with the commonly accepted notion that NTD is dispensable for VLP production [18], [19], but rather plays a role in the rearrangement of different Gag components during the maturation process. We are currently investigating the reasons why the CA NTD appears to be important in VLP production at least under our experimental settings. It may be that the NTD region allows weak interactions with the CTD during virus assembly as suggested by a previous study [31]. The authors clearly demonstrated that point mutations in helices 4–6 greatly reduce viral production. It is also possible that these mutations disrupt the overall folding of CA. The structure of full-length HIV-1 CA has been previously depicted by Ganser-Pornillos and colleagues [10], [64] and it was shown that NTD-NTD forms the hexamerisation interface, whilst CTD-CTD forms the dimerization interface, and that an additional interface exists between NTD and CTD in a mature CA context. This might help to explain why deletion of the CA NTD prevents VLP formation.

In conclusion, the observed requirement of NTD for Gag-Gag interaction and consequently for Gag assembly may explain the failure of Gag min to lead to VLP formation in our experimental setting. Therefore, we believe that a better understanding of mechanisms underlying VLP production will help the scientific community in future design of VLP-based HIV-1 vaccines.

## Methods

### Pr55^Gag^ molecular constructs

Gag sequences were derived from the hGag-TAP construct (i.e. HXB2-based vector) (GenBank K03455) in which codons were humanized [65]. Gag fragments were PCR amplified with Taq Hi-Fi (Invitrogen, Burlington, Canada) from the hGag-TAP construct and cloned into the pcDNA3.1 (+) vector (Invitrogen, Burlington, Canada) using *HindIII* (forward) and *EcoRI* (reverse) restriction sites present in respective PCR primers (restriction sites are underlined in the following primer sequences). We constructed a series of mutant Pr55^Gag^ expression plasmids as depicted in Fig. 1. In brief, the **Gag wild-type (Gag WT)** sequence was amplified using forward primer 5′-CCC AAG CTT ATG GGC GCC CGC GCC AGC and reverse primer 5′-CCG GAA TTC TCA TTG TGA CGA GGG GTC GCT GC. The **MAΔ8**–**132** construct involved deletion of the MA domain except for the myristylation signal sequence included in the forward primer (shown in italic): 5′- CCC AAG CTT *ATG GGC GCC CGC GCC AGC GTG* CCC ATC GTG CAG AAC ATC CAG. The reverse primer was the same one as for the Gag WT construct. In the **p6_449_LD** construct, the p6 domain was replaced by a LD sequence derived from the RSV p2b domain [48], which consists of a TASAPPPPYVG motif in the expressed protein. The corresponding motif sequence was introduced in the reverse primer (shown in italic). The forward primer used was the same as for Gag WT construct, and the reverse primer was 5′- CCG GAA TTC TCA *GCC CAC GTA GGG AGG TGG AGG GGC GCT GGC GGT* AAA ATT CCC TGG CCT TCC CTT G. For the **MAΔ44**–**132** construct, fragment 1 was amplified using the forward primer used for the Gag WT construct. The reverse primer contained the sequences corresponding to the beginning of the CA domain (underlined in the text) and to the end of the basic region sequence (shown in italic): 5′- CTG GAT GTT CTG CAC GAT GGG *GCG CTC CAG CTC GCG GCT G* . For fragment 2, the forward primer contained the end of the basic region domain sequence (shown in italic) and the beginning of the CA domain sequence (underlined in the text): 5′- C *AGC CGC GAG CTG GAG CGC* CCC ATC GTG CAG AAC ATC CAG . The reverse primer was the same one as for the Gag WT construct. The two fragments were combined in a fusion PCR reaction (see Δp6 construct below) using forward and reverse primers used for the Gag WT construct. For the **NCp6_378_LZLD** construct, fragment 1 was PCR amplified with the forward primer used for Gag WT construct. The reverse primer contained the sequence corresponding to the beginning of the LZ domain (underlined in text) from the CREB gene and the end of the SP1 spacer (shown in italic): 5′- TTT CTT CTT TCT ACG ACA CTC TCG *CAT GAT GGT GGC GCT GTT GGT C* . For fragment 2, the DNA template used was the plasmid KCREB1 provided generously by Dr. R. H. Goodman (Vollum Institute, Portland, USA). The forward primer contained the end of the SP1 sequence (shown in italic) and the beginning of the LZ sequence (underlined in the text): 5′- *G ACC AAC AGC GCC ACC ATC ATG* CGA GAG TGT CGT AGA AAG AAG AAA . The reverse primer contained the sequence corresponding to the end of LZ region (underlined in the text), a sequence coding for LD (shown in italic) humanized according to a previously published code [50] and the restriction site for *EcoRI* (underlined in the text): 5′- CCG GAA TTC TCA *GCC CAC GTA GGG AGG TGG AGG GGC GCT GGC GGT* ATC TGA TTT GTG GCA GTA AAG G . The two fragments were combined in a fusion PCR (see Δp6 construct below) using the forward primer used for fragment 1 amplification and the reverse primer used for fragment 2 amplification. For the **CAΔNTD** construct, fragment 1 was amplified using the forward primer used for the Gag WT construct. The reverse primer contained the sequences corresponding to the beginning of the CA MHR domain (underlined in the text) and to the end of the MA domain (shown in italic): 5′- GTC CAG GAT GCT GGT GGG GCT *GTA GTT CTG GCT CAC CTG GTT G* . For fragment 2, the forward primer contained the end of the MA domain (shown in italic) and the beginning of the CA MHR domain (underlined in the text): 5′- *C AAC CAG GTG AGC CAG AAC TAC* AGC CCC ACC AGC ATC CTG GAC . The reverse primer was the one used for the Gag WT construct. The 2 fragments were combined in a fusion PCR reaction (see Δp6 construct below) using forward and reverse primers used for the Gag WT construct.

The **Gag min** construct was based on published work describing the minimal Gag sequence required to generate VLPs [18]. Fragment 1 was amplified with a forward primer containing a *HindIII* restriction site (underlined in the text), a myristylation site (shown in italic) and the beginning of the CA MHR domain (underlined in the text): 5′- CCC AAG CTT *ATG GGC GCC CGC GCC AGC GTG* AGC CCC ACC AGC ATC CTG GAC . The reverse primer contained the sequences corresponding to the beginning of LZ domain (underlined in the text) and to the end of the SP1 sequence (shown in italic): 5′- TTT CTT CTT TCT ACG ACA CTC TCG *CAT GAT GGT GGC GCT GTT GGT C* . For fragment 2, the template DNA used was the KCREB1 plasmid. The forward primer contained sequences corresponding to the end of SP1 (underlined in the text) and to the beginning of the LZ domain (shown in italic): 5′- G ACC AAC AGC GCC ACC ATC ATG *CGA GAG TGT CGT AGA AAG AAG AAA* . The reverse primer was the same one as for the NCp6_378_LZLD construct. The 2 fragments were combined in a fusion PCR using the forward primer used for fragment 1 and the reverse primer used for fragment 2.

For both **Δp6** and **ΔNCp6** constructs, the forward primer was the same one as for the Gag WT construct. The reverse primer used for the Δp6 construct was 5′- CCG GAA TTC TCA AAA ATT CCC TGG CCT TCC CTT G. The reverse primer used for the ΔNCp6 construct was 5′- CCG GAA TTC TCA CAT GAT GGT GGC GCT GTT GG. Chimeric sequences were generated by fusion PCR. Briefly, two fragments were PCR amplified using Taq Hi-Fi. The reverse primer of fragment 1 contained a sequence for future annealing to the beginning of fragment 2 and the forward primer of fragment 2 contained a sequence for future annealing to the end of fragment 1. Both PCR products were purified on DNA-binding columns (Qiagen, Mississauga, Canada). Then, 2.5 µl of each purified fragment were combined with a master mix containing Taq Hi-Fi buffer, MgSO4, dNTPs and Taq Hi-Fi. Five cycles of PCR amplification were completed under these conditions. During the denaturation step of cycle 5, primers were added: a forward primer containing the *HindIII* restriction site annealing to the beginning of fragment 1, and a reverse primer containing the *EcoRI* restriction site annealing with the end of fragment 2. Next, 25–30 additional cycles were completed. The fusion PCR product was purified on agarose gel, then digested with *HindIII* and *EcoRI* and cloned into vector pcDNA3.1 (+).

### Cell culture and transfection

The HEK293T cell line was used to produce VLP stocks. This cell line was obtained from the American Type Culture Collection (Manassas, VA) and maintained in Dulbecco's modified Eagle medium (DMEM) (Invitrogen, Burlington, Canada) supplemented with 10% heat-inactived foetal bovine serum (Invitrogen, Burlington, Canada), penicillin G (100 U/ml), and streptomycin (100 µg/ml) at 37°C in 5% CO_2_. For calcium phosphate transfection, the various plasmids were transfected in 40–50% confluent HEK293T cells in T75 cm^2^ flasks (BD, Oakville, Canada), as described previously [66], [67]. Briefly, fifteen micrograms of plasmid were used to transfect HEK293T cells and at 6 h post-transfection, the medium was discarded and cells washed twice with phosphate-buffered saline (PBS). Finally, 10 ml of fresh medium was added and the cells were incubated for an additional 48 hours.

### Preparation of cell lysates and VLP supernatants

Cell supernatants were collected 48 h post-transfection, centrifuged at 4°C for 5 min at 300 × g, and filtered through a 0.22 µM pore-size cellulose acetate membrane to remove cellular debris (ThermoFisher Scientific, Ottawa, Canada). For virus release assays, the cell-free supernatants were centrifuged through a 2 ml 20% sucrose cushions in TSE (10 mM Tris hydrochloride [pH 7.5], 100 mM NaCl, 1 mM EDTA) at 4°C for 45 min at 274,000 × g (SW41 rotor at 40,000 rpm; 2 ml of cushion for 10 ml of supernatant from each T75 cm^2^ flask) [21]. The pellets were then resuspended in 150 µl of immunoprecipitation buffer (IPB) (20 mM Tris hydrochloride [pH 7.4], 50 mM NaCl, 1% Triton X-100) containing a protease inhibitor cocktail (ThermoFisher Scientific, Ottawa, Canada). The samples were aliquoted and stored at −80°C. As for the transfected cells, they were washed once with 10 ml of room temperature PBS, detached with 10 ml of ice-cold PBS, and pelleted with a brief centrifugation at 4°C for 5 min at 300 × g. The cell pellets were lysed in 1 ml of IPB containing protease inhibitors, followed by a brief vortexing and microcentrifugation at 4°C for 5 min at 13,000 × g to remove insoluble materials. Cell lysates were aliquoted and stored at −80°C. All cell lysate samples were normalized with BCA protein quantification assays (Thermo Fisher Scientific, Ottawa, Canada).

### Gradient SDS-PAGE and Coomassie blue staining

Normalized quantities of cell lysates or VLP supernatants were mixed with an equal volume of 2X sample buffer (125 mM Tris hydrochloride [pH 6.8], 4% sodium dodecyl sulfate (SDS), 20% glycerol, 3.1% dithiothreitol (DTT), 0.25% bromophenol blue), and boiled for 5 min at 100°C. Samples were left to migrate on a 7.5–20% gradient SDS-polyacrylamide gel electrophoresis (PAGE). Thereafter, the gels were incubated for 15 min in Coomassie Brilliant Blue (Bio-Rad laboratories, Mississauga, Canada) (0.4% R250, 10% acetic acid, 30% methanol), and then discoloured in a mixture of 10% acetic acid and 30% methanol. Gels were digitized with a scanner (SnapScan e20, AGFA). Benchmark Standard ladder protein was used (Invitrogen, Burlington, Canada).

### Immunoprecipitation (IP) and Western blots analyses

To precipitate the Gag protein, we used the monoclonal antibody specific for the major core capsid protein p24 purified from the 183-H12-5C hybridoma (AIDS Repository Reagent Program, Germantown, USA). Briefly, 5 µg of 183-H12-5C antibody were added to cell lysates or cell-free supernatants at 4°C for at least 30 min on a rotary shaker (Labquake, Thermo scientific, Ottawa, Canada). Next, 30 µl of protein A/G plus-Agarose (Santa Cruz Biotechnology, Santa Cruz, USA) were added, and agitated at 4°C for 1 h on a rotary shaker. Next, four washes were done with IPB, followed by microcentrifugation at 4°C for 30 sec at 13,200 × g. Finally, pellets were resuspended with an equal volume of 2× sample buffer (125 mM Tris hydrochloride [pH 6.8], 4% sodium dodecyl sulfate (SDS), 20% glycerol, 3.1% dithiothreitol (DTT), 0.25% bromophenol blue). After boiling for 5 min, the samples were microcentrifuged for 1 min at 13,000 × g and transferred to a new tube in order to eliminate beads. Samples were left to migrate on gradient SDS-polyacrylamide gel as described above.

After SDS-PAGE migration, proteins were transferred onto Immobilon-P PVDF 0.45 µM membranes (Thermo Fisher Scientific, Ottawa, Canada) by standard blotting technique. Proteins were revealed with a biotinylated anti-p24 antibody (183-H12-5C hybridoma) (EZ-link sulfo-NHS-LC-Biotin Thermo Fisher Scientific, Ottawa, Canada). Primary biotinylated antibodies were detected with Cy-3-conjugated streptavidin (Jackson ImmunoResearch Lab, Inc) at a working concentration of 0.5 µg/ml. Fluorescent signal detection was achieved by scanning the membrane with a fluorescent laser scanner Typhoon 9200 (GE Healthcare Canada, Mississauga, Canada). In order to determine the protein's molecular weight, the standard protein ladder used was the ECL Plex Fluorescent Rainbow markers (GE Healthcare Canada, Mississauga, Canada). HIV-1 Gag proteins immunodetected on membranes were quantitated with ImageQuant version 5.2 software, and background correction (local average) was applied for all conditions tested. Graphic was normalized with Pr55^Gag^ wild-type proteins (WT) and expressed as a percentage of WT.

### Electron microscopy

HEK293T cells were transfected with the studied VLP constructs as described above. The cells were harvested 48 h post-transfection, fixed in a solution of 0.1 M cacodylate buffer containing 8% sucrose and 2.5% glutaraldehyde, washed in the same buffer overnight, and then post-fixed with 1% osmium tetroxide at 4°C for 90 min. Dehydration with graded ethanol was then performed, and the cell samples were embedded in Eponate resin (Poly/Bed 812; Polysciences, Burlington, Canada). Sections of 60–80 nm were stained with uranyl acetate and lead citrate, and observed under a JEOL 1010 TEM at a magnification of 50,000, Digital images were processed with the Gatan digital micrograph software version 3.10.1.

### Indirect immunofluorescence assays

Prior to transfection, cells (1×10^4^) were plated in 24-well plates (BD, Oakville, Canada) containing German Glass coverslips coated with poly-L-Lysine. Cells were transfected using the calcium phosphate protocol described above; 48 h post-transfection, cells were washed twice with PBS, fixed in 4% paraformaldehyde (PFA) at room temperature for 20 min, and washed four times with PBS. Cells were incubated for 30 min with blocking/permeabilization buffer (1% bovine serum albumin (BSA), 0.1% Triton X-100, and 10% human heat inactivated serum (HHIS)). After four additional washes with PBS, cells were stained with the anti-p24 183-H12-5C antibody. Following washes with PBS, cells were incubated with an Alexa 488-conjugated goat anti-mouse secondary antibody (Invitrogen, Burlington, Canada) and, finally, the nuclei were stained with 4′,6-diamidino-2-phenylindole (DAPI) provided by Invitrogen (Burlington, Canada). After several washes, slides were mounted in Fluoromount G (Invitrogen, Burlington, Canada). The samples were visualized using a Nikon eclipse TE 300 inverted microscope at a 1000X magnification. Digital images were processed with ImageJ software, version 1.42q (NIH, rsbweb.nih.gov/ij).

## References

[1] Deml L, Speth C, Dierich MP, Wolf H, Wagner R (2005). Recombinant HIV-1 Pr55gag virus-like particles: potent stimulators of innate and acquired immune responses.. Mol Immunol.

[2] Doan LX, Li M, Chen C, Yao Q (2005). Virus-like particles as HIV-1 vaccines.. Rev Med Virol.

[3] Petry H, Goldmann C, Ast O, Luke W (2003). The use of virus-like particles for gene transfer.. Curr Opin Mol Ther.

[4] Furumoto H, Irahara M (2002). Human papilloma virus (HPV) and cervical cancer.. J Med Invest.

[5] Garland SM, Smith JS (2010). Human papillomavirus vaccines: current status and future prospects.. Drugs.

[6] Kwak K, Yemelyanova A, Roden RB (2011). Prevention of cancer by prophylactic human papillomavirus vaccines.. Curr Opin Immunol.

[7] Cimarelli A, Darlix JL (2002). Assembling the human immunodeficiency virus type 1.. Cell Mol Life Sci.

[8] Adamson CS, Freed EO (2007). Human immunodeficiency virus type 1 assembly, release, and maturation.. Adv Pharmacol.

[9] Adamson CS, Jones IM (2004). The molecular basis of HIV capsid assembly–five years of progress.. Rev Med Virol.

[10] Ganser-Pornillos BK, Yeager M, Sundquist WI (2008). The structural biology of HIV assembly.. Curr Opin Struct Biol.

[11] Wills JW, Craven RC (1991). Form, function, and use of retroviral gag proteins.. AIDS.

[12] Gheysen D, Jacobs E, de Foresta F, Thiriart C, Francotte M (1989). Assembly and release of HIV-1 precursor Pr55gag virus-like particles from recombinant baculovirus-infected insect cells.. Cell.

[13] Briggs JA, Simon MN, Gross I, Krausslich HG, Fuller SD (2004). The stoichiometry of Gag protein in HIV-1.. Nat Struct Mol Biol.

[14] Paliard X, Liu Y, Wagner R, Wolf H, Baenziger J (2000). Priming of strong, broad, and long-lived HIV type 1 p55gag-specific CD8+ cytotoxic T cells after administration of a virus-like particle vaccine in rhesus macaques.. AIDS Res Hum Retroviruses.

[15] Young KR, McBurney SP, Karkhanis LU, Ross TM (2006). Virus-like particles: designing an effective AIDS vaccine.. Methods.

[16] Chukkapalli V, Hogue IB, Boyko V, Hu WS, Ono A (2008). Interaction between the human immunodeficiency virus type 1 Gag matrix domain and phosphatidylinositol-(4,5)-bisphosphate is essential for efficient gag membrane binding.. J Virol.

[17] Zhou W, Parent LJ, Wills JW, Resh MD (1994). Identification of a membrane-binding domain within the amino-terminal region of human immunodeficiency virus type 1 Gag protein which interacts with acidic phospholipids.. J Virol.

[18] Accola MA, Strack B, Göttlinger HG (2000). Efficient particle production by minimal Gag constructs which retain the carboxy-terminal domain of human immunodeficiency virus type 1 capsid-p2 and a late assembly domain.. J Virol.

[19] Borsetti A, Ohagen A, Göttlinger HG (1998). The C-terminal half of the human immunodeficiency virus type 1 Gag precursor is sufficient for efficient particle assembly.. J Virol.

[20] Reil H, Bukovsky AA, Gelderblom HR, Göttlinger HG (1998). Efficient HIV-1 replication can occur in the absence of the viral matrix protein.. EMBO J.

[21] Wang CT, Lai HY, Li JJ (1998). Analysis of minimal human immunodeficiency virus type 1 gag coding sequences capable of virus-like particle assembly and release.. J Virol.

[22] Freed EO, Orenstein JM, Buckler-White AJ, Martin MA (1994). Single amino acid changes in the human immunodeficiency virus type 1 matrix protein block virus particle production.. J Virol.

[23] Göttlinger HG, Sodroski JG, Haseltine WA (1989). Role of capsid precursor processing and myristoylation in morphogenesis and infectivity of human immunodeficiency virus type 1.. Proc Natl Acad Sci U S A.

[24] Dorfman T, Mammano F, Haseltine WA, Göttlinger HG (1994). Role of the matrix protein in the virion association of the human immunodeficiency virus type 1 envelope glycoprotein.. J Virol.

[25] Lodge R, Göttlinger H, Gabuzda D, Cohen EA, Lemay G (1994). The intracytoplasmic domain of gp41 mediates polarized budding of human immunodeficiency virus type 1 in MDCK cells.. J Virol.

[26] Berthet-Colominas C, Monaco S, Novelli A, Sibai G, Mallet F (1999). Head-to-tail dimers and interdomain flexibility revealed by the crystal structure of HIV-1 capsid protein (p24) complexed with a monoclonal antibody Fab.. EMBO J.

[27] Worthylake DK, Wang H, Yoo S, Sundquist WI, Hill CP (1999). Structures of the HIV-1 capsid protein dimerization domain at 2.6 A resolution.. Acta Crystallogr D Biol Crystallogr.

[28] Chazal N, Carriere C, Gay B, Boulanger P (1994). Phenotypic characterization of insertion mutants of the human immunodeficiency virus type 1 Gag precursor expressed in recombinant baculovirus-infected cells.. J Virol.

[29] Hong SS, Boulanger P (1993). Assembly-defective point mutants of the human immunodeficiency virus type 1 Gag precursor phenotypically expressed in recombinant baculovirus-infected cells.. J Virol.

[30] Trono D, Feinberg MB, Baltimore D (1989). HIV-1 Gag mutants can dominantly interfere with the replication of the wild-type virus.. Cell.

[31] von Schwedler UK, Stray KM, Garrus JE, Sundquist WI (2003). Functional surfaces of the human immunodeficiency virus type 1 capsid protein.. J Virol.

[32] Joshi A, Nagashima K, Freed EO (2006). Mutation of dileucine-like motifs in the human immunodeficiency virus type 1 capsid disrupts virus assembly, gag-gag interactions, gag-membrane binding, and virion maturation.. J Virol.

[33] Ono A, Waheed AA, Joshi A, Freed EO (2005). Association of human immunodeficiency virus type 1 gag with membrane does not require highly basic sequences in the nucleocapsid: use of a novel Gag multimerization assay.. J Virol.

[34] Wright ER, Schooler JB, Ding HJ, Kieffer C, Fillmore C, et al. (2007). Electron cryotomography of immature HIV-1 virions reveals the structure of the CA and SP1 Gag shells.. EMBO J.

[35] Abdurahman S, Hoglund S, Goobar-Larsson L, Vahlne A (2004). Selected amino acid substitutions in the C-terminal region of human immunodeficiency virus type 1 capsid protein affect virus assembly and release.. J Gen Virol.

[36] Liang C, Hu J, Russell RS, Roldan A, Kleiman L (2002). Characterization of a putative alpha-helix across the capsid-SP1 boundary that is critical for the multimerization of human immunodeficiency virus type 1 gag.. J Virol.

[37] Liang C, Hu J, Whitney JB, Kleiman L, Wainberg MA (2003). A structurally disordered region at the C terminus of capsid plays essential roles in multimerization and membrane binding of the gag protein of human immunodeficiency virus type 1.. J Virol.

[38] Darlix JL, Godet J, Ivanyi-Nagy R, Fosse P, Mauffret O (2011). Flexible nature and specific functions of the HIV-1 nucleocapsid protein.. J Mol Biol.

[39] Feng YX, Copeland TD, Henderson LE, Gorelick RJ, Bosche WJ (1996). HIV-1 nucleocapsid protein induces "maturation" of dimeric retroviral RNA in vitro.. Proc Natl Acad Sci U S A.

[40] Muriaux D, De Rocquigny H, Roques BP, Paoletti J (1996). NCp7 activates HIV-1Lai RNA dimerization by converting a transient loop-loop complex into a stable dimer.. J Biol Chem.

[41] Khorchid A, Halwani R, Wainberg MA, Kleiman L (2002). Role of RNA in facilitating Gag/Gag-Pol interaction.. J Virol.

[42] Crist RM, Datta SA, Stephen AG, Soheilian F, Mirro J (2009). Assembly properties of human immunodeficiency virus type 1 Gag-leucine zipper chimeras: implications for retrovirus assembly.. J Virol.

[43] Zhang Y, Qian H, Love Z, Barklis E (1998). Analysis of the assembly function of the human immunodeficiency virus type 1 gag protein nucleocapsid domain.. J Virol.

[44] Göttlinger HG, Dorfman T, Sodroski JG, Haseltine WA (1991). Effect of mutations affecting the p6 gag protein on human immunodeficiency virus particle release.. Proc Natl Acad Sci U S A.

[45] VerPlank L, Bouamr F, LaGrassa TJ, Agresta B, Kikonyogo A (2001). Tsg101, a homologue of ubiquitin-conjugating (E2) enzymes, binds the L domain in HIV type 1 Pr55(Gag).. Proc Natl Acad Sci U S A.

[46] Bieniasz PD (2009). The cell biology of HIV-1 virion genesis.. Cell Host Microbe.

[47] Strack B, Calistri A, Craig S, Popova E, Göttlinger HG (2003). AIP1/ALIX is a binding partner for HIV-1 p6 and EIAV p9 functioning in virus budding.. Cell.

[48] Ott DE, Coren LV, Gagliardi TD, Nagashima K (2005). Heterologous late-domain sequences have various abilities to promote budding of human immunodeficiency virus type 1.. J Virol.

[49] Strack B, Calistri A, Göttlinger HG (2002). Late assembly domain function can exhibit context dependence and involves ubiquitin residues implicated in endocytosis.. J Virol.

[50] Wada K, Aota S, Tsuchiya R, Ishibashi F, Gojobori T (1990). Codon usage tabulated from the GenBank genetic sequence data.. Nucleic Acids Res 18 Suppl.

[51] Ako-Adjei D, Johnson MC, Vogt VM (2005). The retroviral capsid domain dictates virion size, morphology, and coassembly of gag into virus-like particles.. J Virol.

[52] Ono A (2010). HIV-1 assembly at the plasma membrane.. Vaccine.

[53] Ono A (2007). Subcellular locations at which HIV-1 assembles.. Uirusu.

[54] Ono A, Ablan SD, Lockett SJ, Nagashima K, Freed EO (2004). Phosphatidylinositol (4,5) bisphosphate regulates HIV-1 Gag targeting to the plasma membrane.. Proc Natl Acad Sci U S A.

[55] Bryant M, Ratner L (1990). Myristoylation-dependent replication and assembly of human immunodeficiency virus 1.. Proc Natl Acad Sci U S A.

[56] Demirov DG, Freed EO (2004). Retrovirus budding.. Virus Res.

[57] Hansen M, Jelinek L, Whiting S, Barklis E (1990). Transport and assembly of gag proteins into Moloney murine leukemia virus.. J Virol.

[58] Jones TA, Blaug G, Hansen M, Barklis E (1990). Assembly of gag-beta-galactosidase proteins into retrovirus particles.. J Virol.

[59] Ono A, Orenstein JM, Freed EO (2000). Role of the Gag matrix domain in targeting human immunodeficiency virus type 1 assembly.. J Virol.

[60] Sandefur S, Varthakavi V, Spearman P (1998). The I domain is required for efficient plasma membrane binding of human immunodeficiency virus type 1 Pr55Gag.. J Virol.

[61] Ono A, Freed EO (2004). Cell-type-dependent targeting of human immunodeficiency virus type 1 assembly to the plasma membrane and the multivesicular body.. J Virol.

[62] Kiernan RE, Ono A, Englund G, Freed EO (1998). Role of matrix in an early postentry step in the human immunodeficiency virus type 1 life cycle.. J Virol.

[63] Yuan X, Yu X, Lee TH, Essex M (1993). Mutations in the N-terminal region of human immunodeficiency virus type 1 matrix protein block intracellular transport of the Gag precursor.. J Virol.

[64] Ganser-Pornillos BK, Cheng A, Yeager M (2007). Structure of full-length HIV-1 CA: a model for the mature capsid lattice.. Cell.

[65] Roy BB, Hu J, Guo X, Russell RS, Guo F (2006). Association of RNA helicase a with human immunodeficiency virus type 1 particles.. J Biol Chem.

[66] Cantin R, Fortin JF, Lamontagne G, Tremblay M (1997). The presence of host-derived HLA-DR1 on human immunodeficiency virus type 1 increases viral infectivity.. J Virol.

[67] Fortin JF, Cantin R, Lamontagne G, Tremblay M (1997). Host-derived ICAM-1 glycoproteins incorporated on human immunodeficiency virus type 1 are biologically active and enhance viral infectivity.. J Virol.

